# Incorporating hierarchical information into multiple instance learning for patient phenotype prediction with single-cell RNA-sequencing data

**DOI:** 10.1093/bioinformatics/btaf241

**Published:** 2025-07-15

**Authors:** Chau Do, Harri Lähdesmäki

**Affiliations:** Department of Computer Science, Aalto University, Espoo 11000, Finland; Department of Computer Science, Aalto University, Espoo 11000, Finland

## Abstract

**Motivation:**

Multiple instance learning (MIL) provides a structured approach to patient phenotype prediction with single-cell RNA-sequencing (scRNA-seq) data. However, existing MIL methods tend to overlook the hierarchical structure inherent in scRNA-seq data, especially the biological groupings of cells or cell types. This limitation may lead to suboptimal performance and poor interpretability at higher levels of cellular division.

**Results:**

To address this gap, we present a novel approach to incorporate hierarchical information into the attention-based MIL framework. Specifically, our model applies the attention-based aggregation mechanism over both cells and cell types, thus enforcing a hierarchical structure on the flow of information throughout the model. Across extensive experiments, our proposed approach demonstrates highly competitive performance and shows robustness against limited sample sizes. Moreover, ablation test results show that simply applying the attention mechanism on cell types instead of cells leads to improved performance, underscoring the benefits of incorporating the hierarchical groupings. By identifying the critical cell types that are most relevant for prediction, we show that our model is capable of capturing biologically meaningful associations, suggesting its potential to facilitate biological discoveries.

**Availability and implementation:**

Our source code is available at https://github.com/minhchaudo/hier-mil. All datasets used in this study are publicly available online.

## 1 Introduction

Single-cell RNA-sequencing (scRNA-seq) has the potential to transform precision medicine by providing high-resolution, disease-specific signatures (He *et al.* 2022). This source of data can improve the prediction of patient-level characteristics, including disease phenotypes and clinical outcomes. During prediction, relevant cells and cell types can be identified, hence supporting biological discoveries (Xiong *et al.* 2023).

In recent years, several methods have been developed for the prediction of patient-level characteristics using scRNA-seq data. Notably, multiple instance learning (MIL) emerges as a promising framework due to its ability to structurally model cellular heterogeneity (Engelmann *et al.* 2024). Specifically, the attention-based aggregation technique has reached state-of-the-art performance as it can model the influence of individual cells on the sample-level prediction in the form of attention weights (Ilse *et al.* 2018). The integration of attention-based MIL with a generalized linear mixed model (GLMM) also reduces the problem of the low signal-to-noise ratio in scRNA-seq data and improves robustness (Engelmann *et al.* 2024).

However, existing MIL models do not account for the hierarchical structure of scRNA-seq data, especially the biological groupings of cells (i.e. cell types). This possibly leads to suboptimal performance, as these models might fail to capture the similarities of cells within a cell type in predicting the phenotype. In addition, the attention-based aggregation technique only offers interpretability at the cell level and not at the cell type level, which makes it difficult to draw associations between cell types and phenotypes. A common workaround is to approximate the cell type contribution as an average of the cell contributions. However, this approach does not account for the heterogeneity of cells within a cell type and therefore might not work well when the proportion of influential cells within a cell type is small. Moreover, after deriving the cell type contributions, existing methods often decide on the critical (i.e. phenotype-driving) cell types by ranking cell types based on their contribution scores and selecting a cut-off value or a top percentile. This method is susceptible to random variations in the data and in model training, making it difficult to ensure the biological and statistical significance of the results.

In this work, we introduce an approach to incorporate hierarchical information into the attention-based MIL framework. Our approach employs existing cell type annotations to perform a step-by-step aggregation procedure, first over cells and subsequently over cell types, enforcing a hierarchical structure on the flow of information throughout the model. Specifically, we propose two aggregation strategies, represented in the cell type attention (CTA) model and the hierarchical attention (HA) model. The CTA model combines mean pooling of cells and attention-based aggregation of cell types, thereby reducing the complexity of scRNA-seq data yet still accounting for the heterogeneity of cell types in predicting the label. Meanwhile, the HA model performs attention-based aggregation of both cells and cell types, thus additionally capturing the heterogeneity of cells in a cell type. The sample-level predictions of both models can be decomposed into a sum of either cell-level or cell type-level contributions, hence providing interpretability at both levels. With the cell type-level contributions directly available, we avoid the need for aggregating the cell-level contributions, thus side-stepping the challenges associated with the heterogeneity and the varying numbers of cells within a cell type. Moreover, we propose using a permutation test to assess the statistical significance of the cell type contributions, thereby ensuring the robust identification of critical cell types. It is worth noting that while we focus our formulation and experiments on cell types, our framework is applicable to any type of cellular groupings, such as cell lineages and clusters.

We benchmark our proposed models against four state-of-the-art models for patient phenotype prediction with scRNA-seq data and one traditional machine learning model. Across different disease phenotype prediction and clinical outcome modeling tasks, our models consistently deliver a competitive performance and demonstrate the ability to recover biologically meaningful associations, underscoring the benefits of implementing a hierarchical design in a MIL framework.

## 2 Related work

### 2.1 Multiple instance learning

MIL refers to a specific setting of weakly supervised learning where data is organized into collections termed *bags*, each containing several *instances* (Ilse *et al.* 2018, Engelmann *et al.* 2024). Note that the number of instances in each bag can vary. The key premise of MIL is that only the bag-level labels are known, and the instance-level labels are unknown (Engelmann *et al.* 2024). The task of MIL models is, therefore, to predict labels of bags using features of their instances (Ilse *et al.* 2018). Given this setup, the problem of predicting phenotypes using scRNA-seq data naturally falls into the scope of MIL, in which bags are the samples from patients/donors, and the instances are the cells.

Ilse *et al.* (2018) introduced the attention-based aggregation technique, which models the bag-level representation as an attention-weighted sum of the instance-level representations. The attention weights are modeled as a transformation of the instance-level representations, followed by a softmax activation over the instances. As the attention weights can be interpreted as the relative importance of instances, this aggregation technique allows MIL models to capture the cellular heterogeneity of scRNA-seq data and also provides interpretability at the single-cell level.

Building upon this work, Engelmann *et al.* (2024) proposed MixMIL, an MIL model that integrates the attention-based aggregation technique with a GLMM. Specifically, MixMIL aggregates the cell representations into the sample representation with the attention technique, then models the dependency between the sample representation and the label with a GLMM. Notably, the attention function is a *shallow* function, consisting of a linear transformation of the cell representation and a softmax over the cells. This design allows MixMIL to simultaneously capture cellular heterogeneity with attention-based MIL and maintain robustness with a GLMM, combining the strengths of both frameworks.

Aside from modeling the relative importance of cells, the attention technique can also be used to capture the interactions between cells. Mao *et al.* (2024) introduced ScRAT, a transformer-based model for patient phenotype prediction using scRNA-seq data. Briefly, the attention mechanism in transformers iteratively updates the representation of a cell as a weighted sum of the representations of all other cells and of itself, hence capturing the influence of cells on one another.

The structured nature of MIL also allows for the incorporation of domain knowledge into the derivation of attention weights. Building upon the assumption that a prior distribution of the instance-level label is available for each instance, Hajj *et al.* (2024) proposed several strategies for prior knowledge incorporation, with the two most successful being attention modulation (AM) and attention training (AT). The AM strategy boosts the derived attention weight of each instance by a factor depending on the (prior) expected label of that instance. On the other hand, AT imposes an additional cross-entropy loss to penalize the discrepancy between the prior and the predicted probability of each instance-level label. This predicted probability is the unnormalized attention weight of each instance followed by sigmoid activation to represent the probability of the instance belonging to the positive class.

### 2.2 Modeling cell subpopulations

Instead of directly applying the MIL framework, other works have attempted to model the cell subpopulations in each scRNA-seq sample and derive their attributes for prediction. CloudPred (He *et al.* 2022) models the distribution of cells with a Gaussian mixture model (GMM) and uses the prevalence of the Gaussian clusters as features for prediction. Instead of directly assigning cells to clusters or assuming a distribution of cells, ProtoCell4P (Xiong *et al.* 2023) directly maintains the representations of cell subpopulations, termed prototypes, in the latent space and regularizes the latent embeddings of cells to cluster around the prototypes. In CloudPred, the parameters of the GMM are initialized with the expectation-maximization method, while the prototypes in ProtoCell4P are initialized randomly. Both frameworks are then optimized end-to-end with respect to the prediction loss via stochastic gradient descent.

## 3 Methodology

### 3.1 Overview

**Figure 1.**
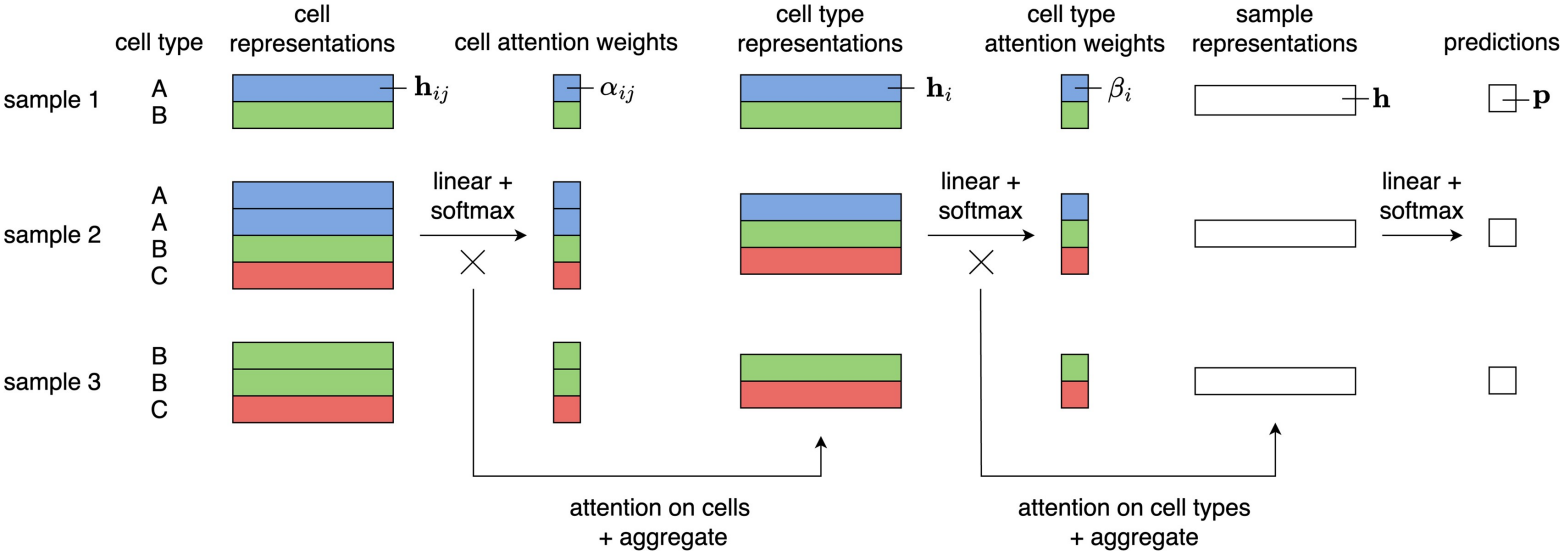
Workflow of the proposed hierarchical attention-based MIL framework for patient phenotype classification using single-cell RNA-sequencing (scRNA-seq) data. The model aggregates cell-level representations and applies attention weights at both the cell and cell type levels to generate a sample-level representation for classification. Note that the derivation of the cell-attention weights $\alpha_{ij}$ in the illustrated workflow is specific for the HA model. For the CTA model, the cell-attention weights $\alpha_{ij}$ are equal for all cells in the same cell type (see Equation (4)).

We present a novel approach to incorporate hierarchical information into an MIL framework for patient phenotype prediction with scRNA-seq data. Our approach processes gene expressions or latent representations of individual cells and performs a two-step aggregation procedure, first over cells and then over cell types to produce sample-level representations for prediction. Note that the first step involves either mean pooling or attention-based aggregation of cells, while the second step performs attention-based aggregation of cell types to produce sample representations. For easy reference, the model with attention only on cell types is termed the CTA model, and the other one with attention on both cells and cell types is termed the HA model. See Fig. 1 for an illustration of our proposed method.

We show that the final prediction can be decomposed into a sum of either cell or cell type contributions, hence providing interpretability at both levels. Based on this, we derive a metric termed importance score that measures the overall contribution of each cell type to the prediction. Moreover, we propose using a permutation test to determine cell types with a significant importance score, which are the ones that might play a role in the mechanism of the patient phenotypes.

### 3.2 Model architecture

We assume to have data from S samples. Data from sample s∈{1,…,S} are denoted as $\left(D_{s},y_{s}\right)$, where $y_{s}\in Y=\{1,\ldots ,C\}$ is the class label and $D_{s}$ denotes the single-cell data. We assume that single cells from each sample are annotated with cell type label i∈{1,…,I}, where I denotes the number of cell types. These annotations can be obtained with any existing cell type annotation tool or can be manually labeled. For each sample s, single-cell data $D_{s}$ consists of gene expression profiles or (possibly lower-dimensional) representations of individual cells and is denoted as $D_{s}=\{x_{\mathrm{sij}}\}$, where $x_{\mathrm{sij}}\in R^{m}$ is the expression profile of the cell $j=1,\ldots ,n_{si}$ with cell type i in sample s (or $x_{\mathrm{sij}}\in Z_{\geq 0}^{m}$ in the case of original gene expression counts). Sample s contains altogether $N_{s}=\sum_{i=1}^{I}n_{si}$ individual cells. Also note that sample s does not necessarily contain all I cell types, i.e. $n_{si}$ can equal zero for some i. For the clarity of notation, we drop the sample index s below and present our method for a single sample, i.e. the single-cell data of a single sample is denoted as $D=\{x_{ij}\}$.

#### 3.2.1 Low-dimensional representations

Before applying the attention mechanism, we pass the input $D=\{x_{ij}\}$ through feed-forward neural network layers (with either one or two hidden layers) to obtain a lower-dimensional representation of each cell, denoted as $h_{ij}\in R^{d}$. For example, for a single hidden layer model, the cell representations are computed using the standard feed-forward model


(1) $$h_{ij}=\varphi (W_{x}x_{ij}+b_{x}),$$

where φ(·) is a chosen non-linearity, such as ReLU, and $W_{x}\in R^{d\times m}$ and $b_{x}\in R^{d}$ are trainable parameters. These cell representations serve as input for all subsequent layers of the neural network, including the two aggregation modules and the classification layer that are described next.

#### 3.2.2 Cell-level aggregation

In this step, the HA model performs an attention-based aggregation over cells. Specifically, for each cell j within cell type i, we compute a cell-level attention score $\alpha_{ij}$ using a linear transformation followed by a softmax function within the cell type:


(2) $$e_{ij}=w^{⊤}h_{ij}+b,\;\alpha_{ij}=\frac{\;\text{exp}(e_{ij})}{\sum_{j^{\prime }=1}^{n_{i}}\;\text{exp}\;(e_{ij^{\prime }})},$$

where $w\in R^{d}$ and b∈R are learnable parameters shared across all cell types, and $\alpha_{ij}$ is normalized over the cells within cell type i. For each cell type i, we then compute an auxiliary cell type representation $h_{i}$ by aggregating the representations of its cells using the cell-level attention weights:


(3) $$h_{i}=\sum_{j=1}^{n_{i}}\alpha_{ij}h_{ij}.$$

Alternatively, in the CTA model, the cell type representation $h_{i}$ is obtained via mean pooling of the cell representations $h_{ij}$:


(4) $$h_{i}=\frac{1}{n_{i}}\sum_{j=1}^{n_{i}}h_{ij}.$$

The factor $\alpha_{ij}$ is therefore defined as $\alpha_{ij}=\frac{1}{n_{i}}$. The CTA model effectively simplifies the cell type representation computation by using equal weights instead of the cell-specific attention weights.

Note that this step is the only difference between the CTA and the HA models. All previous and subsequent steps are described jointly for the two models.

#### 3.2.3 Cell type-level aggregation

At the second level of hierarchy, we compute a cell type-level attention score $\beta_{i}$ using a linear transformation followed by a softmax function over the cell types:


(5) $$e_{i}=v^{⊤}h_{i}+b^{\prime },\;\beta_{i}=\frac{\;\text{exp}(e_{i})}{\sum_{i^{\prime }=1}^{I}\;\text{exp}\;(e_{i^{\prime }})},$$

where $v\in R^{d}$ and $b^{\prime }\in R$ are learnable parameters, and $\beta_{i}$ is normalized over the cell types within a sample.

#### 3.2.4 Sample-level representation

For each sample, we define a sample-level representation by aggregating the auxiliary cell type-level representations using the cell type-level attention weights:


(6) $$\begin{array}{c} h=\sum_{i=1}^{I}\beta_{i}h_{i}=\sum_{i=1}^{I}\beta_{i}\left(\sum_{j=1}^{n_{i}}\alpha_{ij}h_{ij}\right)\\ =\sum_{i=1}^{I}\sum_{j=1}^{n_{i}}\beta_{i}\alpha_{ij}h_{ij}=\sum_{i=1}^{I}\sum_{j=1}^{n_{i}}\gamma_{ij}h_{ij}, \end{array}$$

where we have used Equation (3) and defined


(7) $$\gamma_{ij}=\beta_{i}\alpha_{ij}.$$

Equations (6) and (7) demonstrate that our definition of sample-level representation results in a hierarchical two-step aggregation mechanism, first over the cell representations to obtain the cell type representations, and then over the cell type representations to obtain the sample-level representations. Note that our hierarchical aggregation results in normalized attention weights since $\sum_{i}\sum_{j}\gamma_{ij}=\sum_{i}\beta_{i}\sum_{j}\alpha_{ij}=\sum_{i}\beta_{i}=1$.

#### 3.2.5 Classification layer

A final linear layer maps the representation of each sample to probabilities p.

In the case of multi-class classification, $p={\left[p_{1},\ldots ,p_{C}\right]}^{⊤}$ are the probabilities of the C classes:


(8) p=softmax(Wh+b),

where $W\in R^{C\times d}$ and $b\in R^{C}$ are learnable parameters shared across all samples.

In the case of binary classification, the prediction becomes:


(9) $$p=\text{sigmoid}({\bar{w}}^{⊤}h+\bar{b}),$$

where $\bar{w}\in R^{d}$ and $\bar{b}\in R$ are learnable parameters and p is now a scalar representing the probability of the positive class. The prediction probability vector p can then be written as $p={\left[1-p,\;p\right]}^{⊤}$.

The hierarchical attention-based MIL model described above is applied separately to each of the S samples to obtain sample-specific prediction probabilities $p_{s}={\left[p_{s1},\ldots ,p_{sC}\right]}^{⊤}$. To define a training objective, we employ the cross-entropy loss:


(10) $$L=-\sum_{s=1}^{S}\sum_{c=1}^{C}1(y_{s}=c)\;\text{log}(p_{sc}),$$

where 1(·) is the indicator function.

The objective in Equation (10) is minimized with respect to model parameters $\left(W_{x},b_{x},w,b,v,b^{\prime },W,b,\bar{w},\bar{b}\right)$ using gradient descent.

### 3.3 Model interpretability

Following Engelmann *et al.* (2024), we show that since the classification layer and the pooling function are both linear, the logits of the final prediction readily decompose into a sum of either cell-level or cell type-level contributions. For brevity, we present only the formulations for the multi-class case, but note that the binary case is identical, only with the notations W and b replaced by ${\bar{w}}^{⊤}$ and $\bar{b}$.

Let Wh=z in Equation (8). Using Equation (6), we observe that


(11) $$z=W\sum_{i=1}^{I}\sum_{j=1}^{n_{i}}\gamma_{ij}h_{ij}=\sum_{i=1}^{I}\sum_{j=1}^{n_{i}}\gamma_{ij}(Wh_{ij}).$$

This decomposition highlights that the final logits z+b are a sum of the cell-level logits $\gamma_{ij}Wh_{ij}$, shifted by the bias b. Similarly,


(12) $$z=W\sum_{i=1}^{I}\beta_{i}h_{i}=\sum_{i=1}^{I}\beta_{i}(Wh_{i}),$$

showing that the final logits z+b are a sum of the cell type-level logits $\beta_{i}Wh_{i}$, shifted by the bias b.

### 3.4 Identifying critical cell types

As our models are interpretable at the cell type level, we can identify the *critical cell types* that are most relevant for the prediction and thus likely play a role in the expression mechanism of the disease phenotypes or clinical outcomes.

To identify these critical cell types, we first decompose the prediction logit of each sample into a sum of cell type-level logits. In the case of binary classification, a positive cell type logit indicates contribution to the positive class, while a negative logit represents contribution to the negative class. Hence, on average, cell types with higher logits in positive samples and lower logits in negative samples contribute more to the correct prediction. We introduce a metric termed *importance score* to measure the overall contribution of each cell type:


(13) $$\kappa_{i}=\frac{1}{\left|S_{1}\right|}\sum_{s\in S_{1}}\ell_{si}-\frac{1}{\left|S_{0}\right|}\sum_{s\in S_{0}}\ell_{si}$$

where $\kappa_{i}$ is the importance score of cell type i, $S_{0}$ is the set of negative samples, $S_{1}$ is the set of positive samples, and $\ell_{si}=\beta_{si}{\bar{w}}^{⊤}h_{si}$ is the logit of cell type i in sample s following the decomposition demonstrated in Equation (12). For a formulation of the cell type importance score in the multi-class case, see Supplementary Section S4.1.

We suggest using a permutation test to identify cell types with a statistically significant importance score. Specifically, we repeatedly permute the sample labels, train the model on this permuted data, and compute the cell type importance scores. This results in a null distribution of importance scores for each cell type, from which we can calculate the empirical *P*-values and identify the critical cell types. Note that since we are essentially performing one statistical test for each cell type, multiple testing correction is needed to avoid inflating the false positive rate.

## 4 Experiments and results

### 4.1 Data

We evaluate the proposed models and all baseline models on three scRNA-seq datasets: Cardio (Chaffin *et al.* 2022), COVID (Ziegler *et al.* 2021), and immune checkpoint blockade (ICB) (Gondal *et al.* 2024). The key characteristics of the datasets are given in Table 1. Each dataset is uniformly preprocessed across all models to ensure a consistent comparison of their architectures and training strategies.

**Table 1.** Summary of datasets.

Dataset name	Samples (class distribution)	Avg. cells per sample	Cell types	Classes
Cardio	42 (16 + 15 + 11)	14 111	13	3
COVID	50 (15 + 35)	539	36	2
ICB	57 (38 + 19)	163	23	2

The Cardio dataset published by Chaffin *et al.* (2022) contains single-nucleus expression profiles of patients with dilated and hypertrophic cardiomyopathy. The task we perform on this dataset is to classify the samples based on the patients’ disease status, either dilated cardiomyopathy, hypertrophic cardiomyopathy, or normal (healthy control). Following Xiong *et al.* (2023), to preprocess the data, we removed genes with nonzero expressions in fewer than five cells, normalized the total gene expression counts of each cell to sum up to $10^{4}$, and log-transformed the counts. We then used the scGPT model (Cui *et al.* 2024) with pretrained weights from the whole-human checkpoint to extract the embedding for each cell. Each cell is thus represented by a vector of m=512 dimensions, which serves as input to the models.

The COVID dataset published by Ziegler *et al.* (2021) provides single-cell expression profiles of COVID-19 patients with three disease statuses (COVID-19, long COVID-19, respiratory failure) and of healthy controls. Since the long COVID-19 and respiratory failure classes contain too few samples, samples from these two classes are removed, transforming the task into binary classification. Following Xiong *et al.* (2023), the preprocessing steps involve removing genes with nonzero expressions in fewer than five cells, normalizing the total gene expression counts of each cell to sum up to $10^{4}$, and log-transforming the counts. We then annotate the single cells using the R package singler (Aran *et al.* 2019) with the Human Primary Cell Atlas reference dataset (Mabbott *et al.* 2013). The scGPT model (Cui *et al.* 2024) with pretrained weights from the whole-human checkpoint is then used to embed cells into a m=512 dimensional vector space.

The ICB dataset is a pan-cancer dataset on patients undergoing the ICB treatment. The dataset consists of scRNA-seq samples from eight studies, compiled and preprocessed by Gondal *et al.* (2024). We are using pre-treatment samples from three studies (Bassez *et al.* 2021, Alvarez-Breckenridge *et al.* 2022, Pozniak *et al.* 2024) and two cancer types (melanoma and breast cancer) with different subtypes. The objective is to predict whether patients respond favorably or unfavorably to ICB treatment. The samples are preprocessed and downsampled to at most 200 cells per sample by Gondal *et al.* (2024). Since the original data does not contain cell type annotations, we annotated the cells using the R package singler (Aran *et al.* 2019) with the Blueprint/ENCODE reference dataset (The ENCODE Project Consortium 2012, Martens and Stunnenberg 2013). For this task, we use a set of genes which is a merger of cancer-related genes and immune signatures. Genes missing from the dataset are removed, resulting in a set of m=824 genes. The normalized and log-transformed expressions of these genes are used as input to the models. The list of gene signatures is given in Supplementary Section S1. In addition, we also experiment with using the scGPT-pretrained embeddings of all genes instead of selecting a subset of genes. Specifically, we conduct the repeated, nested cross-validation (CV) experiment described in Section 4.2 with these input representations and report the results in Supplementary Table S4.

### 4.2 Baselines and evaluation

We benchmark our proposed models against four existing methods for phenotype prediction using single-cell data: ScRAT (Mao *et al.* 2024), ProtoCell4P (Xiong *et al.* 2023), CloudPred (He *et al.* 2022), and MixMIL (Engelmann *et al.* 2024). Additionally, we include a commonly used traditional machine learning model, a random forest classifier, which is trained on the mean-pooled representations of the single cells in each sample. All models are evaluated following a repeated, nested 10-fold CV procedure. Specifically, we perform nested CV and calculate the performance metrics, including the area under the receiver operating characteristic curve (AUC), F1 score, accuracy, precision, and recall on the stacked predictions from all folds. The hyperparameters of our models and competing models are optimized using the inner CV loop, such that the combination of hyperparameters that achieves the highest AUC on the stacked predictions from all inner CV folds is used to train the model. This procedure is repeated 10 times with different random seeds for CV splitting, resulting in 10 values for each metric, and the mean and standard deviation of the metrics are reported. For more details on hyperparameter tuning, please see Supplementary Section S2.

The AUC and F1 scores of all seven models on the three benchmark datasets are given in Tables 2 and 3, respectively. The results for the remaining metrics are given in Supplementary Section S3. Our models show highly competitive performance on all datasets across all metrics, demonstrating the utility of our proposed framework. It is worth noting that on the COVID dataset, when using the original annotations by Ziegler *et al.* (2021) instead of the ones obtained with singler, ProtoCell4P achieves an AUC of 0.89±0.02, which is on par with our proposed models.

**Table 2.** Comparison of AUC scores across different models and datasets.^a^

Model	Cardio	COVID	ICB
ScRAT	0.85 ± 0.02	0.83 ± 0.04	0.70 ± 0.06
ProtoCell4P	0.91 ± 0.02	0.85 ± 0.02	0.68 ± 0.04
CloudPred	0.82 ± 0.02	0.82 ± 0.05	0.58 ± 0.05
MixMIL	0.87 ± 0.02	0.83 ± 0.03	0.65 ± 0.06
Random Forest	0.84 ± 0.01	0.84 ± 0.03	0.68 ± 0.05
Our model (CTA)	**0.98** ± **0.01**	**0.90** ± **0.03**	**0.77** ± **0.04**
Our model (HA)	**0.99** ± **0.02**	**0.89** ± **0.03**	**0.75** ± **0.06**

a The two best mean AUCs for each dataset are highlighted in bold.

**Table 3.** Comparison of F1 scores across different models and datasets.^a^

Model	Cardio	COVID	ICB
ScRAT	0.67 ± 0.04	0.73 ± 0.04	**0.63** ± **0.04**
ProtoCell4P	0.72 ± 0.05	0.71 ± 0.05	0.60 ± 0.04
CloudPred	0.52 ± 0.05	0.65 ± 0.05	0.48 ± 0.03
MixMIL	0.70 ± 0.04	0.72 ± 0.03	**0.63** ± **0.05**
Random Forest	0.63 ± 0.04	0.76 ± 0.02	0.53 ± 0.04
Our model (CTA)	**0.91** ± **0.02**	**0.78** ± **0.03**	**0.66** ± **0.05**
Our model (HA)	**0.91** ± **0.04**	**0.77** ± **0.03**	**0.63** ± **0.07**

a The two best mean F1s for each dataset are highlighted in bold.

Also note that our two models, CTA and HA, achieve comparable performance on all benchmark datasets. As the CTA model involves a mean pooling over cells, it assumes equal contribution of cells towards the cell type representation, which proves to be adequate for the tasks at hand. However, on more complex datasets with heterogeneous cell subpopulations in the same cell type, we postulate that the HA model might be more suitable, as it accounts for the variability of both cells and cell types in predicting the label.

### 4.3 Impact of data quality

Sample sizes are typically small in early-stage clinical trials, and biological sampling can result in a limited number of single cells. Moreover, our method relies on existing cell type annotations or cell type annotation methods, which can be inaccurate. To evaluate the robustness of our model against these variations in data quality, we conduct three additional experiments. In the following sections, we present and discuss the results of our experiments on the Cardio dataset. The results on the other datasets can be found in Supplementary Section S5.

#### 4.3.1 Varying train size

**Figure 2.**
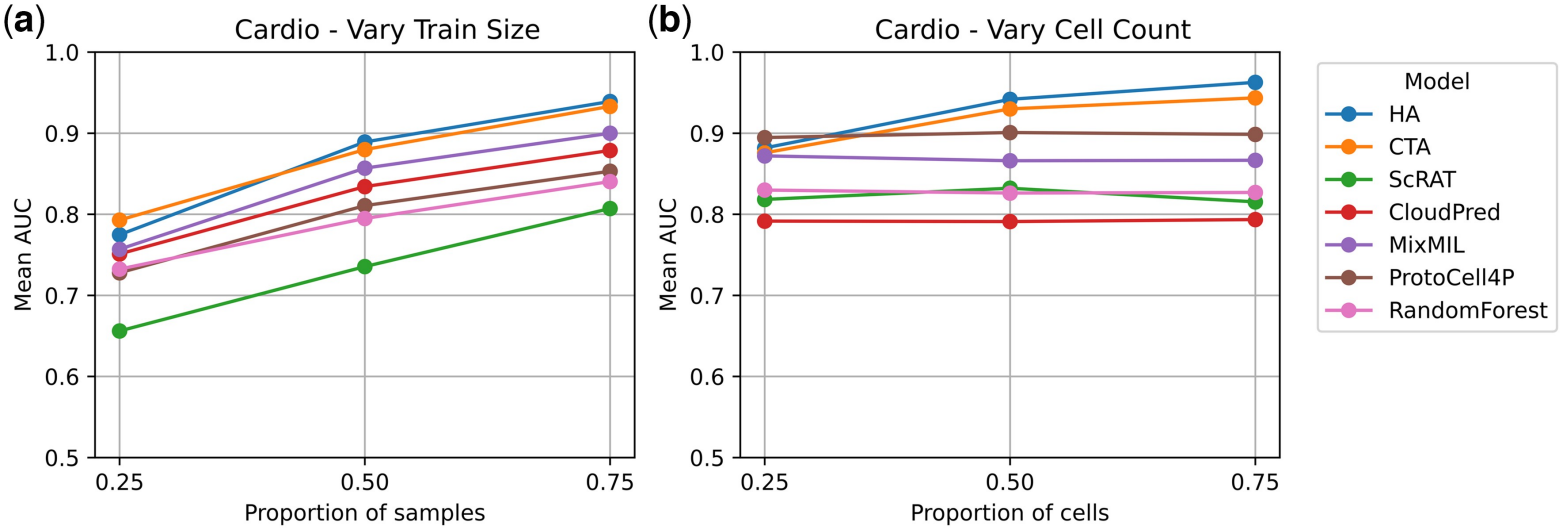
(a) Performance of models on the Cardio dataset when the training sizes are varied. *x*-axis corresponds to the proportion of samples in the original dataset, which for the Cardio dataset is S=42; (b) Performance of models on the Cardio dataset when the cell counts are varied. *x*-axis corresponds to the proportion of cells in each sample.

First, we investigate the impact of limited training size on model performance by sequentially using 25%, 50%, and 75% of the samples for training and reserving the rest for testing. For each training size, we run the experiment for 100 random train-test splits. The AUC is calculated on the testing set at each run, and the mean AUC over 100 runs is reported. This experiment is conducted for all models on all datasets. The results on the Cardio dataset are summarized in Fig. 2a. Results on other datasets are given in Supplementary Fig. S1.

Overall, both of our models demonstrate competitive performance, achieving the highest mean AUC scores across all training sizes on the Cardio and ICB datasets. Notably, our models are capable of achieving reasonable mean AUCs even when trained on only a handful of samples, showing robustness against limited sample sizes.

#### 4.3.2 Varying cell counts

The second experiment studies the effect of varying cell counts on performance. We randomly subsample 25%, 50%, and 75% of the cells in each sample and evaluate the model following the nested CV procedure described in Section 4.2. This process is repeated 10 times for random cell subsampling and CV splitting, resulting in 10 AUCs, and the mean AUC is reported. This experiment is conducted for all models on all except the ICB dataset, since this dataset is already downsampled to at most 200 cells per sample. The results on the Cardio dataset are illustrated in Fig. 2b. The results on the COVID dataset are given in Supplementary Fig. S3.

In general, the performance of our models improves as the proportion of cells in each sample increases, suggesting the ability to effectively utilize the information from additional cells. Moreover, comparing the results of the first and second experiments, all tested models appear less sensitive to the number of cells than to the number of training samples. In fact, the performance of existing methods on both benchmark datasets either plateaus or improves slightly as the proportion of cells increases. These results suggest that the performance of existing methods is generally stable after a certain number of cells are included, consistent with the findings of He *et al.* (2022).

#### 4.3.3 Randomizing cell type annotations

**Figure 3.**
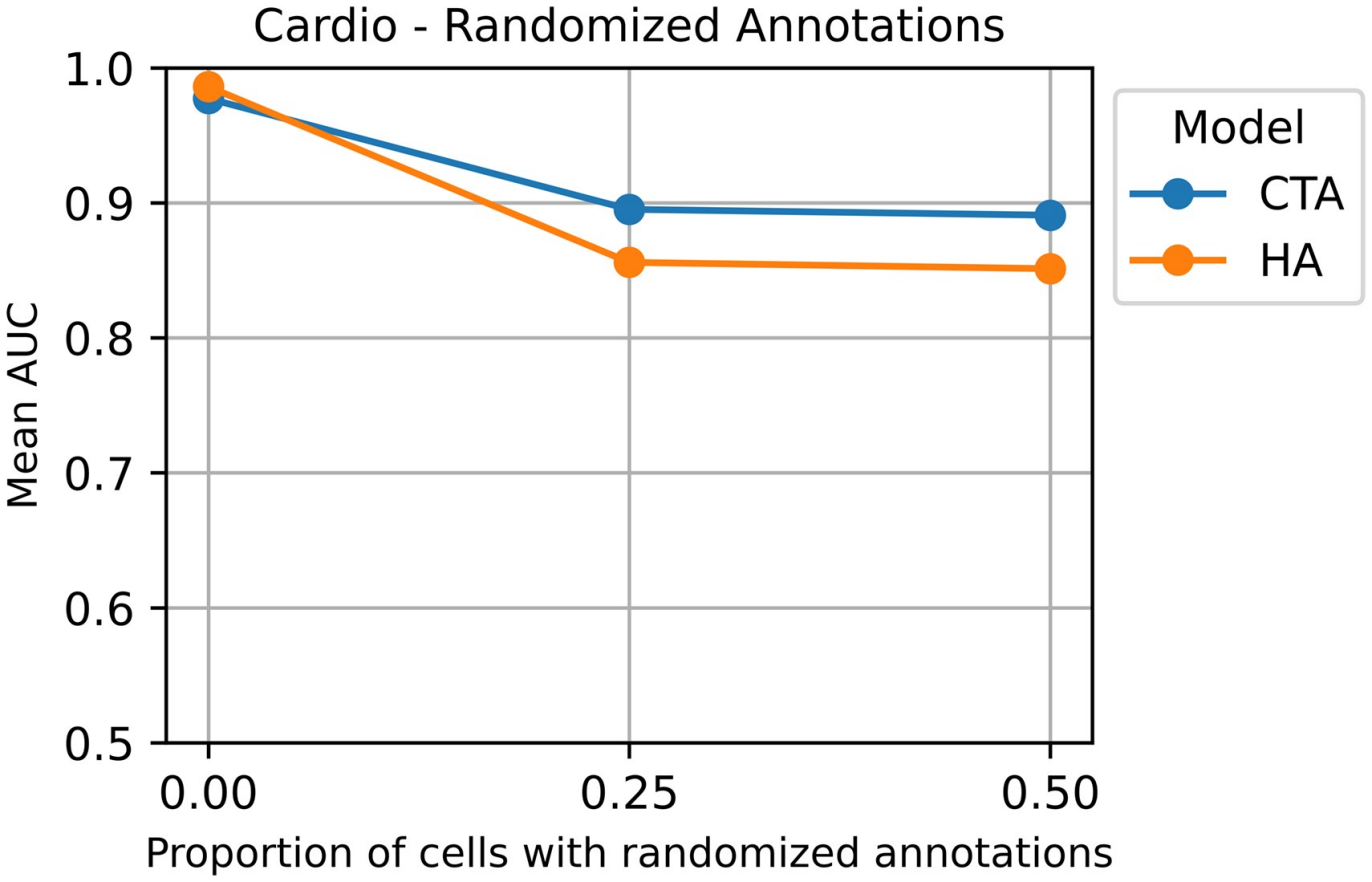
Performance of the proposed models on the Cardio dataset with varying levels of cell type annotation accuracy. *x*-axis corresponds to the proportion of cells that are artificially assigned a randomly chosen cell type annotation.

In the third experiment, we assess the influence of noisy cell type annotations on the performance of our models. We sample 25% and 50% of cells in each sample and assign them random cell types drawn from a uniform distribution over all cell types in the dataset. For each ratio of noisy annotations, we evaluate our models using the nested CV procedure described in Section 4.2. This process is repeated 10 times for random cell sampling, annotation reassignment, and CV splitting, resulting in 10 AUCs, and the mean AUC is reported. Note that this experiment is only conducted for our models, since other models do not require cell type annotations as input (except for ProtoCell4P, which optionally uses cell type annotations). The results on the Cardio dataset are summarized in Fig. 3. Results on other datasets are given in Supplementary Fig. S5. Note that the mean AUCs at 0.0 (no random annotations) are the same as the CV results presented in Table 2.

As our approach relies on existing cell type annotations, the performance of our model declines as the cell type annotations become less accurate. The CTA model appears less sensitive to the quality of the annotations than the HA model, as the mean-pooling operation at the cell level might be more robust to noise compared to the attention-based aggregation operation (Engelmann *et al.* 2024). This dependence on accurate annotations is a potential downside of our approach and can possibly be alleviated with data augmentation strategies. However, results obtained with off-the-shelf cell type annotation tools, such as singler (Aran *et al.* 2019), reported in Table 2 still demonstrate highly competitive performance.

### 4.4 Ablation test

To validate the benefits of incorporating cell type information into the MIL framework, we conduct an ablation test. Specifically, we compare the performance of our two proposed models with two modified models, one with mean pooling over the cells, and the other with attention on cells. Note that the two modified models do not use cell type annotations, thus allowing us to pinpoint the impact of additionally using this information, either through attention on cell types (CTA model) or through attention on both cells and cell types (HA model).

In the mean-pooling model, the entire attention-based aggregation module is omitted, and the sample-level representation is generated by averaging the cell-level representations:


(14) $$\bar{h}=\frac{1}{N}\sum_{i=1}^{I}\sum_{j=1}^{n_{i}}h_{ij}.$$

Here, $h_{ij}$ is the representation of cell j of cell type i, N is the total number of cells in the sample, and $\bar{h}$ is the sample representation. This resembles converting scRNA-seq data to pseudo-bulk representation.

On the other hand, in the cell-attention model, only the cell-type attention layer is omitted, and the sample-level representation is a weighted sum of the cell-level representations:


(15) $${\tilde{e}}_{ij}=w^{⊤}h_{ij}+b,\;{\tilde{\alpha }}_{ij}=\frac{\;\text{exp}({\tilde{e}}_{ij})}{\sum_{i^{\prime }=1}^{I}\sum_{j^{\prime }=1}^{n_{i}}\;\text{exp}\;({\tilde{e}}_{i^{\prime }j^{\prime }})},$$

(16) $$\tilde{h}=\sum_{i=1}^{I}\sum_{j=1}^{n_{i}}{\tilde{\alpha }}_{ij}h_{ij}.$$

Here, ${\tilde{e}}_{ij}$ and ${\tilde{\alpha }}_{ij}$ are the unnormalized and normalized attention weights of cell j of cell type i. This ablation corresponds to the attention mechanism used in MixMIL method (Engelmann *et al.* 2024).

The two modified models (mean pooling and cell attention) are assessed on all three datasets following the repeated, nested CV procedure described in Section 4.2.

The results of the ablation tests are summarized in Table 4.

**Table 4.** Ablation test results.^a^

Model	Cardio	COVID	ICB
Mean pooling	0.91 ± 0.02	**0.90** ± **0.02**	0.74 ± 0.03
Cell attention	0.91 ± 0.03	0.82 ± 0.04	0.70 ± 0.04
Our model (CTA)	**0.98** ± **0.01**	**0.90** ± **0.03**	**0.77** ± **0.04**
Our model (HA)	**0.99** ± **0.02**	0.89 ± 0.03	**0.75** ± **0.06**

a Note that the results for our two proposed models are the same as in Table 2, also presented for easy reference. The two best mean AUCs for each dataset are highlighted in bold.

Our proposed models outperform the two modified models on all except the COVID dataset, where the mean-pooling model also achieves the highest AUC of 0.90±0.02, the same as the CTA model. This result underscores the utility of incorporating cell type information into the attention-based MIL framework for modeling scRNA-seq data. Notably, the performance edge of the CTA model over the cell-attention model suggests the benefits of applying the attention mechanism on cell types instead of cells.

One surprising result is that the mean-pooling model performs relatively better than the cell-attention model on all datasets. One possible explanation for this could be that the mean-pooling operation offers reduced complexity, which helps with the small sample size and the low signal-to-noise ratio of scRNA-seq data. Also note that the cell-attention model does not equate to the MixMIL model. Despite having the same attention mechanism applied on cells, MixMIL lacks the dimensionality reduction input layers and uses a variational inference framework.

### 4.5 Biological relevance

To investigate the interpretability of our approach, we identify the cell types that are critical for the HA model in the COVID and the Cardio datasets following the permutation testing procedure described in Section 3.4. Moreover, we validate the critical cell types against existing literature, thereby assessing the model’s ability to capture biologically relevant associations. For more details on the implementation of the permutation test, see Supplementary Section S4.2. The analysis on the COVID data is presented below, while the analysis on the Cardio data can be found in Supplementary Section S4.3. Additionally, we evaluate and discuss the biological relevance and interpretability of MixMIL and ProtoCell4P in Supplementary Sections S6.1 and S6.2, respectively.

Note that in this experiment, for the COVID dataset, we use the original cell type annotations by Ziegler *et al.* (2021) as they are more fine-grained than the annotations obtained with singler. Table 5 provides the cell type importance scores and their adjusted *P*-values. At the significance level of .05, our model identifies 11 cell types as critical in distinguishing between normal and COVID samples: macrophages, ciliated cells, secretory cells, B cells, goblet cells, developing secretory and goblet cells, plasmacytoid DCs (pDCs), basal cells, T cells, squamous cells, and deuterosomal cells. These findings generally align with the disease mechanism of COVID-19 and the biological changes in response to infection.

**Table 5.** Cell type importance scores and adjusted *P*-values in the COVID dataset.^a^

Cell type	Importance score	Adjusted *P*-value
**Macrophages**	0.17	**.05**
**Ciliated cells**	0.13	**.00**
**Secretory cells**	0.11	**.00**
**B cells**	0.09	**.05**
**Goblet cells**	0.06	**.00**
Developing ciliated cells	0.06	.07
**Developing secretory and goblet cells**	0.06	**.05**
**Plasmacytoid DCs**	0.05	**.05**
**Basal cells**	0.05	**.05**
Mitotic basal cells	0.05	.06
**T cells**	0.05	**.05**
Ionocytes	0.05	.06
**Squamous cells**	0.04	**.05**
Dendritic cells	0.04	.09
**Deuterosomal cells**	0.04	**.05**
Erythroblasts	0.02	.06
Mast cells	0.01	.14
Enteroendocrine cells	−0.02	.64

a The critical cell types with significant adjusted *P*-values (P≤.05) are highlighted in bold.

Ciliated cells in the nasal respiratory epithelium are known to be among the primary sites of viral replication, especially at the onset of COVID-19 (Ahn *et al.* 2021). Infection in these cells can damage the ciliated layer and impair the mucociliary clearance function (Robinot *et al.* 2021). Upon the loss of the ciliated layer, basal cells undergo proliferation and differentiation into ciliated cells as part of the repair mechanism of the epithelium (Robinot *et al.* 2021). There has been some evidence indicating that secretory and goblet cells are also infection sites, though less prominent than ciliated cells (Zhu *et al.* 2020, Robinot *et al.* 2021). The significance of the immune cells (macrophages, B cells, T cells, pDCs) might reflect their over-activation and possible exhaustion during the immune response to infection (Alahdal and Elkord 2022). Among these, pDCs are considered essential during antiviral immune defense, producing interferons to regulate responses to viral infection (Ngo *et al.* 2024).

## 5 Discussion

In this work, we introduce two strategies to incorporate hierarchical information into the attention-based MIL framework for patient phenotype classification with scRNA-seq data. Specifically, we propose using an attention mechanism that either operates on cell types or both cells and cell types, with the latter accounting for the heterogeneity of both cells and cell types in contributing to the prediction. Through extensive experiments, we demonstrate the robustness and utility of our proposed models, even in data-constrained scenarios. Interestingly, empirical results show that applying the same attention mechanism on cell types instead of cells improves predictive performance, which validates the benefits of utilizing a hierarchical structure. Our approach provides interpretability at both the cell and cell type levels, thus potentially supporting biological discoveries.

As a possible area for improvement, we note that our attention mechanism is relatively simple. Even though this design choice suits the typically small sample size of real-world scRNA-seq datasets, for larger datasets, more complex attention mechanisms (e.g. the attention mechanism in transformers) could be used to enhance the modeling capacity. In addition, we note that our approach can be easily adapted to account for patient-level covariates, simply by extending the final classification layer to accommodate the additional inputs. Our HA structure can also be extended to account for more complex cellular hierarchies, such as lineage relationships between cell types, by adding additional aggregation layers. Moreover, domain knowledge about possible phenotype-driving cells or cell types, if available, can be readily integrated into our framework via the strategies proposed by Hajj *et al.* (2024). These techniques have the potential to improve model performance and facilitate the retrieval of biologically meaningful instances, especially when the proportion of phenotype-driving cells or cell types is expected to be low, or when cellular signatures are subtle. It is also worth experimenting with different data augmentation strategies to improve model robustness, including cell subsampling or sample mixup (Mao *et al.* 2024). Finally, gene set enrichment analysis or pathway analysis methods can be performed on the critical cells and cell types retrieved by the model, allowing for the exploration of biological processes and markers associated with the phenotypes (Subramanian *et al.* 2005, Nguyen *et al.* 2019).

This study and the proposed methods have several limitations. As mentioned in Section 4.3.3, since our models input cell type annotations, noisy annotations can affect model performance. This problem can potentially be alleviated by adopting data augmentation strategies. In addition, model performance when encountering novel cell types during test time should be investigated by future studies to ensure generalizability.

## Supplementary Material

btaf241_Supplementary_Data

## Data Availability

The Cardio dataset is available at https://singlecell.broadinstitute.org/single_cell/study/SCP1303. The COVID dataset is available at https://singlecell.broadinstitute.org/single_cell/study/SCP1289. The ICB dataset is available at https://zenodo.org/records/10407126.
