# Supplementary material for: Incorporating hierarchical information into multiple instance learning for patient phenotype prediction with single-cell RNA-sequencing data
Source: Bioinformatics. 2025 Jul 15;41(Suppl 1):i96–i104. doi: 10.1093/bioinformatics/btaf241 (PMC12261414; doi:10.1093/bioinformatics/btaf241)
Supplement: btaf241_Supplementary_Data [file btaf241_supplementary_data.pdf]

Incorporating Hierarchical Information into Multiple Instance Learning for Patient Phenotype Prediction with scRNA-seq Data: Supplementary Information

Chau Do and Harri Lähdesmäki

December, 2024

1 Genes for ICB treatment response prediction

The list of genes used in the ICB dataset is a merger of immune gene signatures and cancer-related genes. The signatures include:

- Stem.Sig (Zhang et al. 2022)
- IFN-gamma and expanded immune signature (Ayers et al. 2017)
- LRRC15-CAF signature (Dominguez et al. 2020)
- Cytolytic activity signature (Rooney et al. 2015)
- NLPR3 inflammasome signature (Ju et al. 2020)
- TIR signature (Mei et al. 2021)
- TIME signature (Lee et al. 2020)
- Immune signatures (HLA (Liu et al. 2018), TILs (Massink et al. 2015), and IFN response(Yoshihara et al. 2013))
- Immune checkpoint genes (Ju et al. 2020)

A file containing the full list of genes can be found in our repository `https://github.com/minhchaudo/hier-mil`.

2 Details on hyperparameter tuning

Hyperparameter tuning is performed using cross validation. Specifically, for each pair of training and testing sets, we perform cross validation on the training data with different combinations of hyperparameters. The AUC is calculated on the stacked predictions from the cross validation folds, resulting in one AUC value for each hyperparameter combination. We use the hyperparameter combination with the highest AUC to re-train the model on the entire training set, and this final model is used for prediction on the test set.

The hyperparameter search space for each model is given below:

- ScRAT:
  - Learning rate: {1e-4, 1e-3, 1e-2}
  - Number of epochs: {100}
  - Number of attention heads: {1, 2, 4}
  - Dropout rate: {0.0, 0.3, 0.5, 0.7}
  - Weight decay: {1e-4, 1e-3, 1e-2}
  - Whether data augmentation is performed: {True, False}
  - Embedding dimension: {8, 32, 64}
  - Number of augmented samples: {100}

    - PCA: {False} (since dimensionality reduction or gene selection has been performed – please see Section 4.1 in the main text for more details)

- ProtoCell4P:
    - Learning rate: {1e-4, 1e-3, 1e-2}
    - Number of epochs: {50, 75, 100}
    - Output dimension of linear layers in encoder and decoder: {32, 64, 128}
    - Hidden dimension: {8, 16, 32}
    - Number of prototypes: {8, 16, 32}
    - Number of epochs in pretraining: {75}
- CloudPred:
    - Learning rate in pretraining: {1e-2}
    - Number of epochs in pretraining: {100}
    - Learning rate: {1e-2, 1e-3, 1e-4}
    - Number of epochs: {100}
    - Number of centers: {2, 8, 16}
- MixMIL:
    - Number of epochs: {100, 500, 1000}
    - Learning rate: {1e-2, 1e-3, 1e-4}
- Random Forest Classifier:
    - Number of estimators: {50, 100, 200}
    - Maximum tree depth: {3, 5, 10, None}
    - Minimum samples to split a node: {2, 5}
- Our models (CTA and HA):
    - Number of epochs: {100, 500, 1000}
    - Dropout rate: {0.0, 0.3, 0.5, 0.7}
    - Weight decay: {1e-4, 1e-3, 1e-2}
    - Hidden dimensions: {32, 64, 128}
    - Number of linear input layers: {1, 2}
    - Learning rate: {5e-3, 1e-3}

Other hyperparameters are kept at their default values.

Since the hyperparameter search spaces of our models, ScRAT, and ProtoCell4P are larger, we perform Bayesian hyperparameter tuning with the Python package `optuna` (Akiba et al. 2019). The number of trials is set to 30. For other models, grid search is used. We note that the hyperparameter search spaces and the number of tuning trials are chosen to balance search coverage and computational resource availability.

3 Results of cross validation experiments

The accuracy, precision, and recall of all models in the cross validation experiment described in Section 4.2 of the main text is given in Table 1, Table 2, and Table 3, respectively. Notice that both of our models demonstrate highly competitive performance, consistently achieving the highest or second-highest results on all datasets across all metrics.

Additionally, Table 4 provides the AUCs of all models on the ICB dataset when using scGPT-pretrained embeddings as input. This serves as a comparison to the results obtained using a subset of relevant genes (Section 1), which are provided in Table 2 of the main text. Notice that using the gene subset results in substantially better performance for all models, suggesting that for this dataset, the gene subset is more informative than the embeddings in predicting the label.

Table 1: Comparison of accuracy across different models and datasets. The two best mean accuracies for each dataset are highlighted in bold.

Model	Cardio	COVID	ICB
ScRAT	0.69 ± 0.03	0.75 ± 0.04	0.69 ± 0.04
ProtoCell4P	0.74 ± 0.04	0.78 ± 0.03	0.68 ± 0.02
CloudPred	0.57 ± 0.04	0.77 ± 0.03	0.68 ± 0.02
MixMIL	0.71 ± 0.03	0.74 ± 0.03	0.65 ± 0.05
RF	0.64 ± 0.04	0.81 ± 0.02	0.67 ± 0.03
Our model (CTA)	**0.92 ± 0.02**	**0.83 ± 0.02**	**0.74 ± 0.03**
Our model (HA)	**0.92 ± 0.04**	**0.82 ± 0.02**	**0.73 ± 0.04**

Table 2: Comparison of precision across different models and datasets. The two best mean precisions for each dataset are highlighted in bold.

Model	Cardio	COVID	ICB
ScRAT	0.68 ± 0.04	0.72 ± 0.04	0.65 ± 0.06
ProtoCell4P	0.73 ± 0.05	0.75 ± 0.05	0.63 ± 0.03
CloudPred	0.62 ± 0.05	0.77 ± 0.06	**0.73 ± 0.15**
MixMIL	0.71 ± 0.03	0.73 ± 0.03	0.63 ± 0.04
Random Forest	0.64 ± 0.04	0.78 ± 0.02	0.60 ± 0.07
Our model (CTA)	**0.91 ± 0.02**	**0.81 ± 0.04**	**0.73 ± 0.04**
Our model (HA)	**0.92 ± 0.03**	**0.80 ± 0.04**	0.71 ± 0.09

Table 3: Comparison of recall across different models and datasets. The two best mean recalls for each dataset are highlighted in bold.

Model	Cardio	COVID	ICB
ScRAT	0.68 ± 0.04	0.76 ± 0.05	0.63 ± 0.04
ProtoCell4P	0.72 ± 0.05	0.70 ± 0.05	0.60 ± 0.03
CloudPred	0.54 ± 0.04	0.64 ± 0.04	0.53 ± 0.02
MixMIL	0.72 ± 0.03	**0.77 ± 0.03**	**0.65 ± 0.05**
Random Forest	0.64 ± 0.04	0.75 ± 0.02	0.55 ± 0.03
Our model (CTA)	**0.91 ± 0.02**	**0.77 ± 0.03**	**0.65 ± 0.05**
Our model (HA)	**0.92 ± 0.03**	0.76 ± 0.03	0.62 ± 0.05

Table 4: AUCs of models on the ICB dataset when using scGPT pretrained embeddings as input.

Model	AUC
ScRAT	0.47 ± 0.06
ProtoCell4P	0.51 ± 0.05
CloudPred	0.39 ± 0.06
MixMIL	0.48 ± 0.07
Random Forest	0.55 ± 0.02
Our model (CTA)	**0.63 ± 0.04**
Our model (HA)	**0.64 ± 0.04**

4 Implementation of the model interpretability analysis

4.1 Importance score for multi-class classification

Consider a single sample s. As detailed in Eq. 8 and Eq. 12 of the main text, the sample representation $\mathbf{h}_s$ is matched to the class probabilities $\mathbf{p}_s = [p_1, \ldots, p_C]^\top$ of the C classes with

$$\mathbf{p}_s = \text{softmax}(\mathbf{W}\mathbf{h}_s + \mathbf{b}) = \text{softmax}\left(\sum_{i=1}^{I} \beta_{si}\mathbf{W}\mathbf{h}_{si} + \mathbf{b}\right) = \text{softmax}\left(\sum_{i=1}^{I} \boldsymbol{\ell}_{si} + \mathbf{b}\right) \tag{1}$$

where $\mathbf{h}_s$ is the representation of sample s, $\mathbf{h}_{si}$ is the representation of cell type i in sample s, β_{si} is the attention weight of cell type i in sample s, $\mathbf{W} \in \mathbb{R}^{C\times d}$ and $\mathbf{b} \in \mathbb{R}^C$ are learnable parameters, and we define $\boldsymbol{\ell}_{si} = \beta_{si}\mathbf{W}\mathbf{h}_{si}$, $\boldsymbol{\ell}_{si} \in \mathbb{R}^C$ as the logit vector of cell type i in sample s.

The overall contribution of cell type i in the accurate classification of class k samples is quantified as

$$\kappa_i^{(k)} = \frac{1}{|S_k|} \sum_{s \in S_k} \left(\boldsymbol{\ell}_{si}^{(k)} - \sum_{h \in \{1,\dots,C\} \setminus \{k\}} \boldsymbol{\ell}_{si}^{(h)} \right) \tag{2}$$

where $\kappa_i^{(k)}$ is the importance score of cell type i in class k, S_k is the set of all samples in class k, and $\boldsymbol{\ell}_{si}^{(k)}$ is the kth element of the logit vector of cell type i in sample s.

The class-specific cell type importance scores $\kappa_i^{(c)}$ can be summarized into an overall importance score κ_i,

$$\kappa_i = \sum_{k=1}^{C} \kappa_i^{(c)}, \tag{3}$$

which quantifies the importance of cell type i in classifying all samples into their correct classes.

4.2 Implementation of the permutation test

4.2.1 COVID data

We first perform five-fold cross validation (CV) and stack the predictions from all folds to obtain the predictions for the entire dataset. The importance score for each cell type can then be calculated from the predictions by using Eq. 13 in the main text. The null distribution of importance scores is obtained with 100 permutations. Multiple testing correction is performed with the Benjamini-Hochberg method.

4.2.2 Cardio data

Similar to the procedure for the COVID data, we perform five-fold CV and stack the predictions from all folds to obtain the predictions for the entire dataset. The class-specific and overall importance scores are then calculated by following Eq. 2 and Eq. 3. The null distribution of the overall importance scores is obtained with 1000 permutations, as we observe that using 100 permutations gives unstable estimates for the empirical p-values, especially after multiple testing correction. Multiple testing correction is performed with the Benjamini-Hochberg method.

4.2.3 Hyperparameters for the five-fold cross validation (CV)

In the five-fold CV step, to ensure that the model has properly converged, we use the combination of hyperparameters that is most frequently selected across all outer folds of the repeated, nested CV experiment described in the Section 4.2 of the main text. The same hyperparameters are also used to train the model on the permuted data. However, for the COVID dataset, note that we are using the more fine-grained annotations by Ziegler et al. (2021) in this interpretability analysis rather than the ones obtained with `singler`. As a result, the hyperparameters from the repeated, nested CV experiment might not be directly applicable. Therefore, in the five-fold CV step, we use the default hyperparameters of our model, which have been verified to yield decent predictive performance on this dataset. In general, we recommended using the hyperparameters that yield good predictive performance to ensure proper model convergence and avoid overfitting, thereby allowing the critical cell types to be identified more accurately.

4.3 Interpretability analysis on Cardio data

Table 5 summarizes the class-specific importance score, the overall importance score, and the adjusted p-values of the cell types in the Cardio dataset. At the significance level of 0.05, our method identifies two critical cell types, macrophage and cardiac muscle cell, which play a significant role in separating the three classes. In addition, by looking at the class-specific importance scores, we can identify the most important cell types that contribute to the accurate classification of samples belonging to each class. In the hypertrophic cardiomyopathy class, macrophage is the most important cell type with the highest importance score of 6.86. Meanwhile, in the dilated cardiomyopathy class, cardiac muscle cell is the most important cell type with the importance score of 6.17.

These findings generally align with existing knowledge. According to Yu et al. (2023), macrophages play a pivotal role in inflammatory response and tissue repair, protecting the cardiac tissues from adverse remodeling in hypertrophic cardiomyopathy. On the other hand, cardiac muscle cell is the primary cell type affected in dilated cardiomyopathy. In dilated cardiomyopathy, cardiac muscle cells from both ventricles become dilated and dysfunctional, leading to the progressive stretching and thinning of the myocardium and thus the widening of the ventricular chambers (Mitrut et al. 2018).

However, we note that besides macrophages and cardiac muscle cells, there are other cell types involved in these conditions as well, such as cardiac fibroblasts (Tsuru et al. 2023) and lymphocytes (Zhang et al. 2024).

Therefore, we hypothesize that in this particular dataset, macrophages and cardiac muscle cells play a major role in differentiating between the conditions such that they alone sufficiently explain the label, leading to our model considering only them as critical. To test this hypothesis, we tried using only macrophages and cardiac muscle cells to predict the label and obtained a strong AUC of 0.93 ± 0.03 (the AUC when using all cell types is 0.99 ± 0.02), supporting our hypothesis.

Table 5: Class-specific importance scores, overall importance scores, and adjusted p-values for cell types in the Cardio dataset. The cell types with significant adjusted p-values are in bold.

**Cell Type**	**Normal**	**Hypertrophic Cardiomyopathy**	**Dilated Cardiomyopathy**	**Importance Score**	**Adjusted p-value**
**Macrophage**	13.38	6.86	-1.64	18.61	**0.00**
**Cardiac Muscle Cell**	-0.44	-0.51	6.17	5.22	**0.03**
Mast Cell	0.03	-0.69	2.19	1.53	0.36
Fat Cell	-0.22	0.08	1.18	1.04	0.36
Cardiac Neuron	-0.02	-0.36	1.18	0.80	0.33
Mesothelial Cell	0.00	0.03	0.61	0.64	0.39
Endothelial Cell of Lymphatic Vessel	-0.01	0.21	0.12	0.33	0.39
Endocardial Cell	-0.01	0.18	0.01	0.17	0.39
Vascular Associated Smooth Muscle Cell	-0.01	0.03	0.10	0.11	0.39
Pericyte Cell	-0.01	0.03	0.09	0.11	0.39
Cardiac Ventricle Fibroblast	-0.01	0.00	0.09	0.09	0.39
Cardiac Endothelial Cell	0.00	0.25	-0.18	0.06	0.47
Lymphocyte	0.01	0.09	-0.05	0.05	0.48

5 Results of experiments on varying data quality

For each experiment, we provide two versions of figures. The first version displays the mean AUC, which clearly highlights the general trend of model performance as different aspects of data quality are varied. For completeness, we also include a second version where the standard errors of the AUC estimates are visualized as error bars, and the x values are slightly jittered to avoid the error bars overlapping. It is worth noting that in these experiments, different aspects of data quality are randomly varied, which naturally results in large standard errors. This effect is most notable in the *Varying train size* experiment due to the limited sample sizes of all datasets.

5.1 Varying train size

Figure 1 illustrates the performance of all models when the proportion of training samples is varied. Figure 2 additionally provides the standard error of each data point.

5.2 Varying cell count

Figure 3 shows the performance of all models with various configurations of cell counts. Figure 4 additionally shows the standard error of each data point.

5.3 Randomizing cell type annotations

Figure 5 summarizes the performance of our proposed models when cell type annotations are partly randomized. Figure 6 additionally provides the standard error of each data point.

6 Interpretability analysis for other models

In this section, we investigate the interpretability and biological relevance of MixMIL and ProtoCell4P on the COVID dataset by identifying the cell types that are considered significant for prediction by each model. However, it is not possible to evaluate or compare the results quantitatively, since there lacks a concrete ground truth for the significant cell types in each dataset. Thus, evaluation is based on qualitative alignment with existing biological knowledge. Moreover, unlike our use of the permutation test, MixMIL and ProtoCell4P do not establish a procedure to systematically distinguish critical from non-critical cell types. Instead, the importance or contribution scores provide a metric to quantify the importance of each cell type relative to the others, and the most important cell types can be reported by selecting a cut-off value or top-percentile of scores.

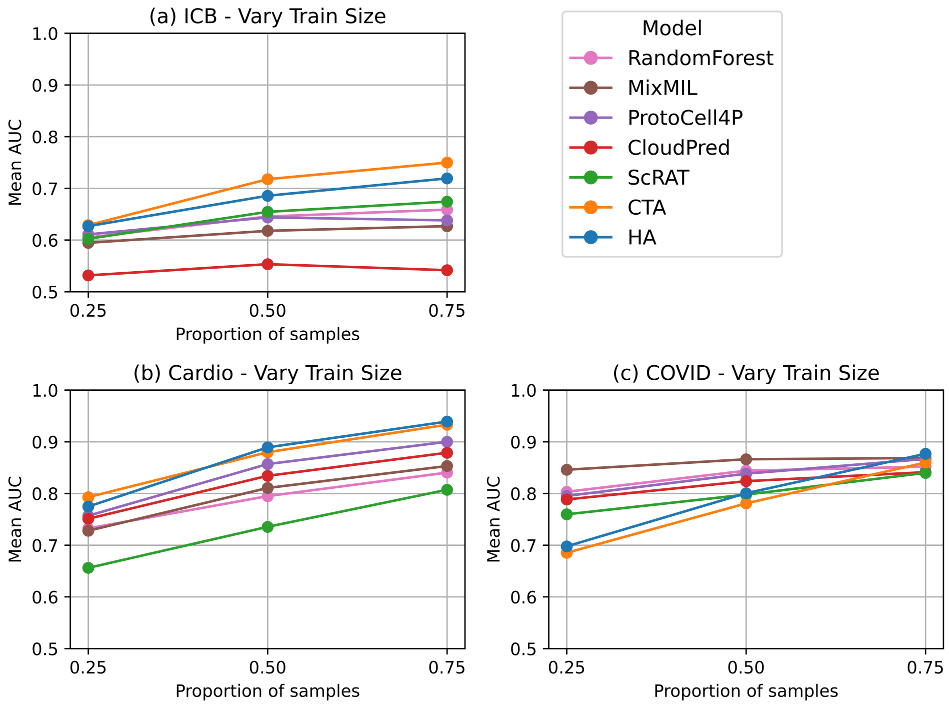

Figure 1: Performance of proposed and existing models on three datasets ((a) - ICB, (b) - Cardio, (c) - COVID) across different proportions of training samples.

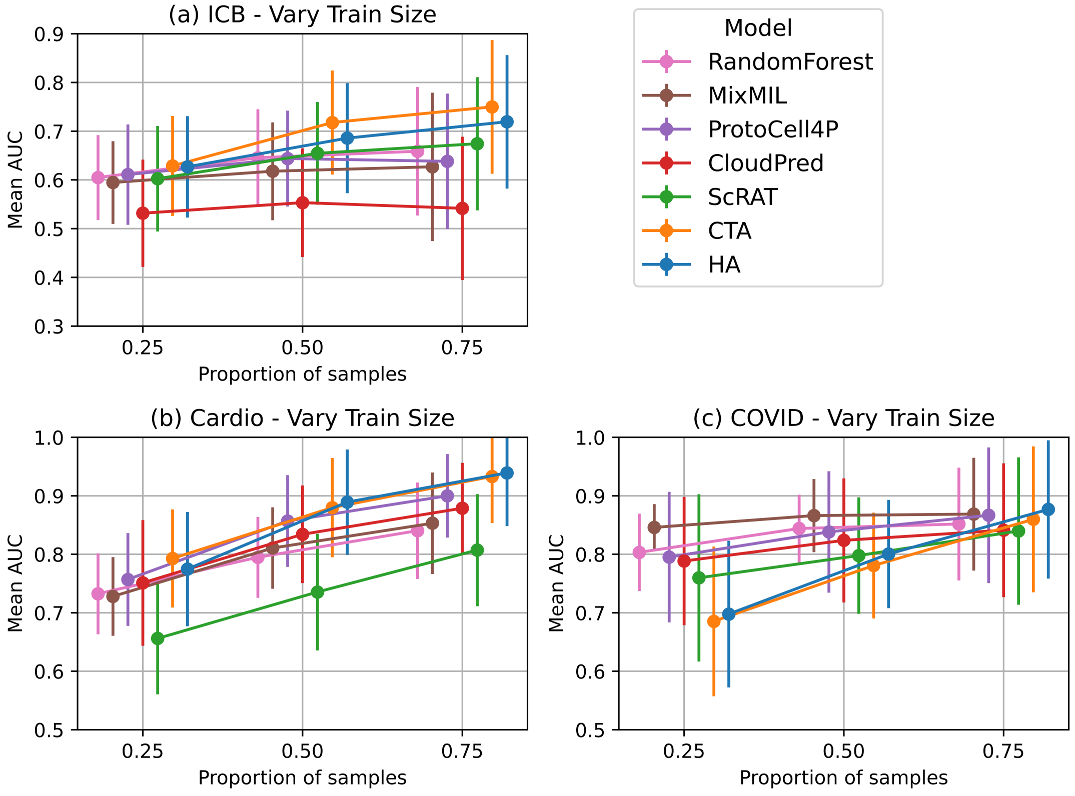

Figure 2: Performance of proposed and existing models on three datasets ((a) - ICB, (b) - Cardio, (c) - COVID) across different proportions of training samples, with error bars included. The x values of the data points are jittered to avoid the overlap of the error bars.

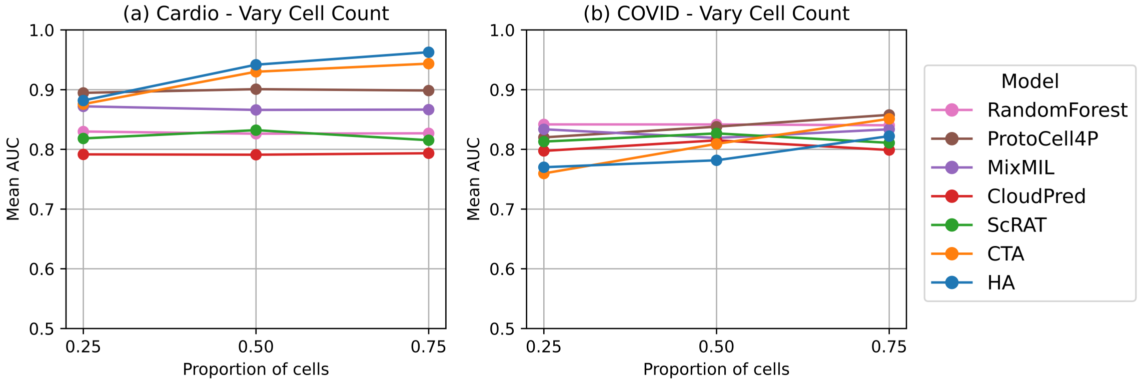

Figure 3: Performance of proposed and existing models on two datasets ((a) - Cardio, (b) - COVID)) across different proportions of cells in each sample.

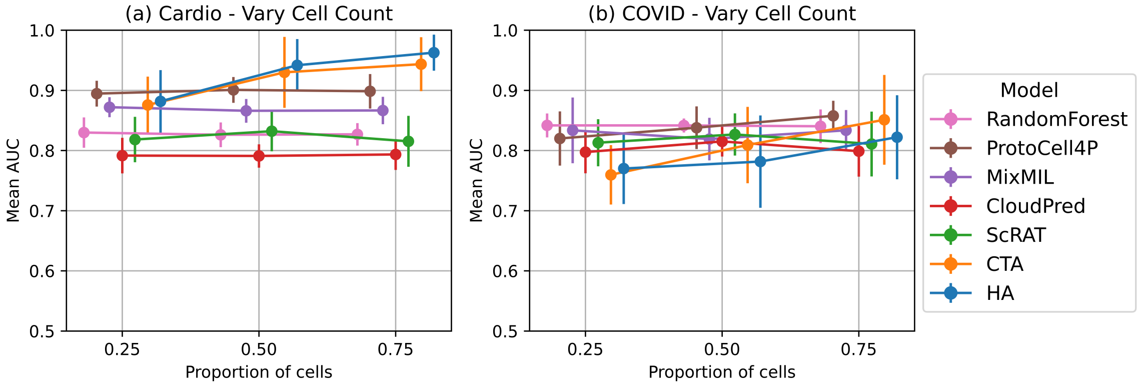

Figure 4: Performance of proposed and existing models on two datasets ((a) - Cardio, (b) - COVID)) across different proportions of cells in each sample, with error bars included. The x values of the data points are jittered to avoid the overlap of the error bars.

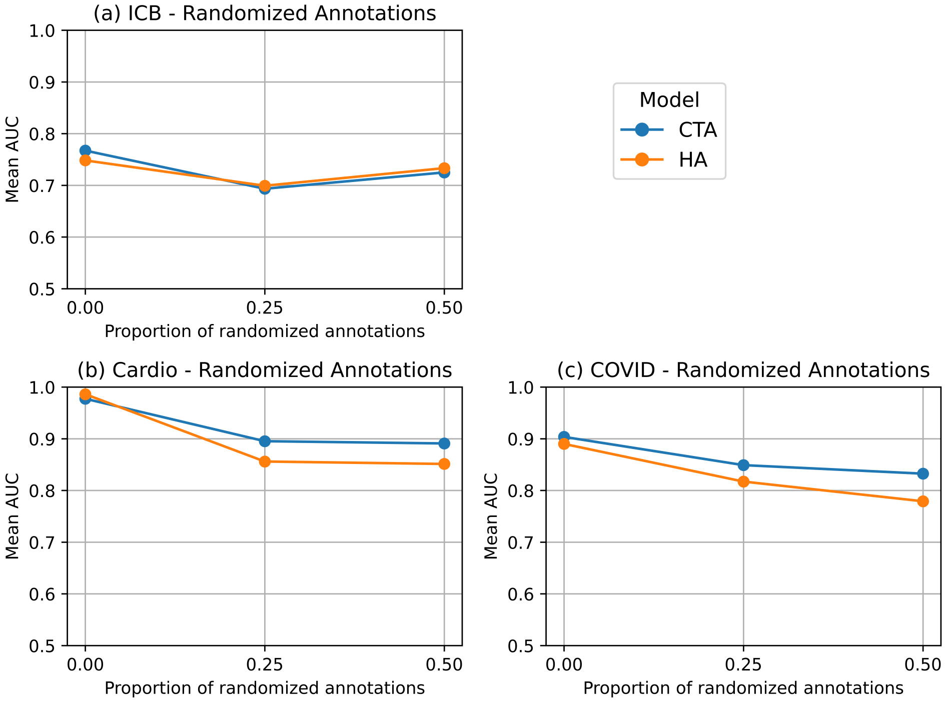

Figure 5: Performance of proposed models on three datasets ((a) - ICB, (b) - Cardio, (c) - COVID) across different proportions of randomized cell type annotations.

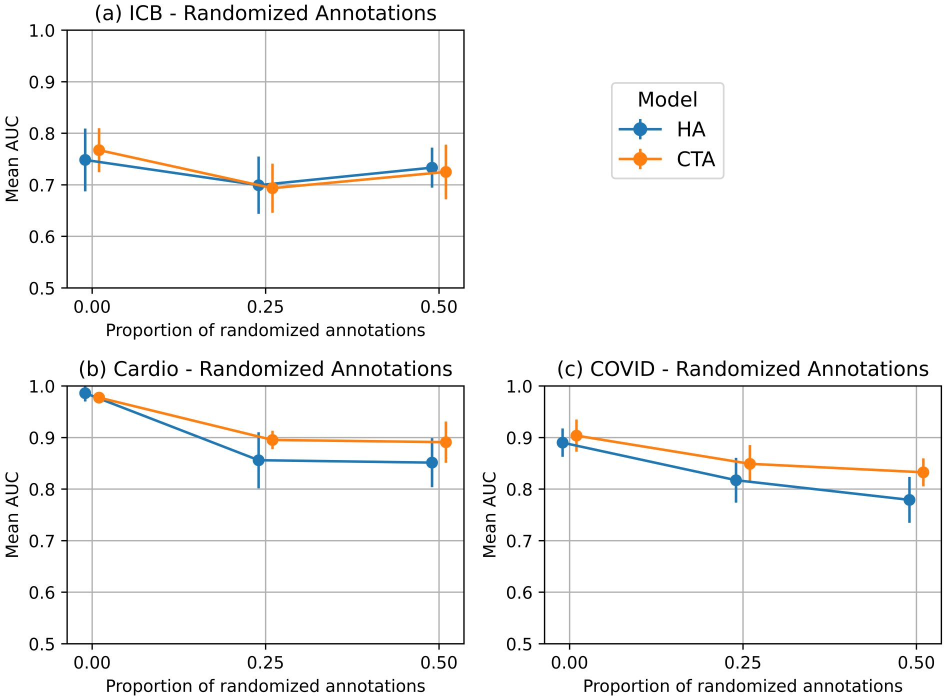

Figure 6: Performance of proposed models on three datasets ((a) - ICB, (b) - Cardio, (c) - COVID) across different proportions of randomized cell type annotations, with standard errors included. The x values of the data points are jittered to avoid the overlap of the error bars.

6.1 MixMIL (Engelmann et al. 2024)

In the case of binary classification (like in the COVID dataset), for each cell j of cell type i in sample s, MixMIL provides a scalar attention weight α_{sij} that represents the relative importance of the cell in the sample. Note that the attention weights are normalized by sample:

$$\sum_i \sum_j \alpha_{sij} = 1 \tag{4}$$

To analyze the interpretability of MixMIL on this dataset, we first perform five-fold cross CV, gather the predictions from all folds, and extract the attention weight α_{sij} of each cell in each sample. The combination of hyperparameters used for five-fold CV is the most frequently selected combination across all outer folds in the repeated, nested CV experiment described in Section 4.2 of the main text.

We define the overall importance ι of cell type i as

$$\iota_i = \frac{1}{S} \sum_{s=1}^{S} \frac{1}{n_{si}} \sum_{j=1}^{n_{si}} \alpha_{sij} \tag{5}$$

where S is the number of samples (patients) and n_{si} is the number of cells with cell type i in sample s.

The relative importance of the cell types in the COVID dataset as quantified using MixMIL is summarized in Table 6. Overall, the high-important cell types generally align with the critical cell types identified by our HA model and with existing literature (for more details, please see Section 4.5 in the main text). However, we note the relatively high importance of enteroendocrine cells, which are typically found in the gastrointestinal tract and likely do not play a significant role in nasal COVID-19. In contrast to MixMIL, our model correctly assigned this cell type a low importance score and considered it insignificant.

6.2 ProtoCell4P (Xiong et al. 2023)

An analysis of the biological interpretability of ProtoCell4P on the COVID dataset has been conducted by Xiong et al. (2023). The three cell types with the highest contribution scores and thus considered most important for the classification of COVID samples are mast cells (3.25), secretory cells (2.60), and goblet cells (2.20). However, we note that ciliated cells, which are supposed to display notable changes during COVID-19 (Ziegler et al. 2021), get a relatively low (absolute) contribution score of 0.83. In contrast, our model accurately detected the importance of ciliated cells and assigned this cell type the second-highest importance score.

Table 6: Importance of cell types in the COVID dataset as identified by MixMIL

**Cell Type**	**Importance**
Ciliated Cells	0.006140
Goblet Cells	0.005703
Developing Ciliated Cells	0.004539
Enteroendocrine Cells	0.004254
Secretory Cells	0.004036
Basal Cells	0.003772
T Cells	0.003751
Squamous Cells	0.003675
Deuterosomal Cells	0.003423
Ionocytes	0.002954
Mitotic Basal Cells	0.002608
Macrophages	0.002337
Developing Secretory and Goblet Cells	0.002190
B Cells	0.001126
Dendritic Cells	0.000757
Erythroblasts	0.000439
Plasmacytoid DCs	0.000229
Mast Cells	0.000137

References

T. Akiba et al. Optuna: A next-generation hyperparameter optimization framework. In *Proceedings of the 25th ACM SIGKDD International Conference on Knowledge Discovery & Data Mining*, KDD '19, pages 2623–31. Association for Computing Machinery, July 2019. doi: 10.1145/3292500.3330701.

M. Ayers et al. Ifn-–related mrna profile predicts clinical response to pd-1 blockade. *The Journal of Clinical Investigation*, 127(8):2930–40, Aug. 2017. doi: 10.1172/JCI91190.

C. X. Dominguez et al. Single-cell rna sequencing reveals stromal evolution into lrrc15+ myofibroblasts as a determinant of patient response to cancer immunotherapy. *Cancer Discovery*, 10(2):232–53, Feb. 2020. doi: 10.1158/2159-8290.CD-19-0644.

J. P. Engelmann et al. Mixed models with multiple instance learning. In *Proceedings of The 27th International Conference on Artificial Intelligence and Statistics*, volume 238 of *Proceedings of Machine Learning Research*, pages 3664–72. PMLR, May 2024.

M. Ju et al. Pan-cancer analysis of nlrp3 inflammasome with potential implications in prognosis and immunotherapy in human cancer. *Briefings in Bioinformatics*, 22(4):bbaa345, Nov. 2020. doi: 10.1093/bib/bbaa345.

H.-S. Lee et al. Comprehensive immunoproteogenomic analyses of malignant pleural mesothelioma. *JCI Insight*, 3(7), Sept. 2020. doi: 10.1172/jci.insight.98575.

Z. Liu et al. A comprehensive immunologic portrait of triple-negative breast cancer. *Translational Oncology*, 11(2):311–29, Apr. 2018. doi: 10.1016/j.tranon.2018.01.011.

M. P. G. Massink et al. Genomic profiling of chek2*1100delc-mutated breast carcinomas. *BMC Cancer*, 15(1): 877, Nov. 2015. doi: 10.1186/s12885-015-1880-y.

Y. Mei et al. A four-gene signature predicts survival and anti-ctla4 immunotherapeutic responses based on immune classification of melanoma. *Communications Biology*, 4(1):1–12, Mar. 2021. doi: 10.1038/s42003-021-01911-x.

R. Mitrut, A. Stepan, and D. Pirici. Histopathological aspects of the myocardium in dilated cardiomyopathy. *Current Health Sciences Journal*, (3):243–9, July 2018. doi: 10.12865/CHSJ.44.03.07.

M. S. Rooney et al. Molecular and genetic properties of tumors associated with local immune cytolytic activity. *Cell*, 160(1):48–61, Jan. 2015. doi: 10.1016/j.cell.2014.12.033.

H. Tsuru et al. Pathogenic roles of cardiac fibroblasts in pediatric dilated cardiomyopathy. *Journal of the American Heart Association*, 12(13):e029676, July 2023. doi: 10.1161/JAHA.123.029676.

G. Xiong, S. Bekiranov, and A. Zhang. Protocell4p: an explainable prototype-based neural network for patient classification using single-cell rna-seq. *Bioinformatics*, 39(8):btad493, Aug. 2023. doi: 10.1093/bioinformatics/btad493.

K. Yoshihara et al. Inferring tumour purity and stromal and immune cell admixture from expression data. *Nature Communications*, 4(1):2612, Oct. 2013. doi: 10.1038/ncomms3612.

Q. Yu et al. Macrophage-specific nlrc5 protects from cardiac remodeling through interaction with hspa8. *JACC: Basic to Translational Science*, 8(5):479–96, May 2023. doi: 10.1016/j.jacbts.2022.10.001.

J. Zhang et al. Immune cells and related cytokines in dilated cardiomyopathy. *Biomedicine Pharmacotherapy*, 171:116159, Feb. 2024. doi: 10.1016/j.biopha.2024.116159.

Z. Zhang et al. Integrated analysis of single-cell and bulk rna sequencing data reveals a pan-cancer stemness signature predicting immunotherapy response. *Genome Medicine*, 14(1):45, Apr. 2022. doi: 10.1186/s13073-022-01050-w.

C. G. Ziegler et al. Impaired local intrinsic immunity to sars-cov-2 infection in severe covid-19. *Cell*, 184(18): 4713–33.e22, Sept. 2021. doi: 10.1016/j.cell.2021.07.023.
